# Evaluation of the laparoscopic component of GESEA Programme in two different groups: Obstetrics and Gynaecology Residents versus Participants in the Annual GESEA Diploma Course in Clermont Ferrand, France

**Published:** 2020-08-05

**Authors:** B Bustos, R Avilés, S Paracchini, B Pereira, R Botchorishvili, B Rabischong

**Affiliations:** Service de Gynecologie-Obstetrique et Medecine de la Reproduction, CHU Estaing, Clermont-Ferrand, France;; Centre International de Chirurgie Endoscopique (CICE), Clermont Ferrand, France;; Universidad de los Andes. Monseñor Álvaro del Portillo, Santiago, Las Condes, Región Metropolitana, Chile / Hospital Parroquial de San Bernardo, San Bernardo, Región Metropolitana, Chile;; Universidad Finis Terrae, Providencia, Región Metropolitana, Chile / Hospital El Carmen, Maipú, Región Metropolitana, Chile;; Department of Surgical Sciences, Azienda Ospedaliera Universitaria Citta della Scienza e della Salute di Torino, Torino, Italy;; Délégation à la Recherche Clinique et à l’Innovation, CHU Estaing. Clermont-Ferrand, France.

**Keywords:** Surgical education, GESEA Certification, Minimally Invasive Surgery, Laparoscopic training courses, Practical skills

## Abstract

**Background:**

Structured laparoscopic training courses are important in surgical education. Different programmes have been proposed, but there is currently no evidence available comparing the performance of specialists versus residents in Obstetrics and Gynaecology at these courses.

**Objective:**

To evaluate the impact of the laparoscopic component of Gynaecological Endoscopic Surgical Education and Assessment (GESEA) Training and Certification courses in two different populations.

**Materials and methods:**

Prospective cohort study. Two groups were analysed - participants of the Residents’ Courses and participants of the Annual Francophone GESEA Diploma Course. Both groups were evaluated using the GESEA Level 1 laparoscopic standardised exercises and carried out in the International Center of Endoscopic Surgery (CICE), Clermont Ferrand, France in 2019.

**Results:**

57 French residents and 69 participants of the Annual GESEA Diploma were evaluated. The average age of participants in the Residents’ Course was lower than those in the Annual Diploma Course (28.4±1.6 versus 35.2±8.0 years, p<0.001). Residents had higher previous experience in laparoscopic surgery (42% vs 36%, p< 0.001), in animal model surgery and in laparoscopic training box (67% vs 36% and 93% vs 67% respectively, p<0.001). Notable improvement was noted in both groups in the camera navigation exercise; first attempt 105±19 vs 117±9 seconds and final attempt 81±15 and 103±20 seconds respectively (p<0.001).

**Conclusions:**

Both groups improved significantly in most of the tests evaluated. French residents had better results in all evaluations, except in one aspect of the suture exercise (maintaining optimal results in performing the knot). After excluding the residents who attended the Annual Diploma Course, all the differences between both groups were statistically more significant.

## Introduction

For many years and in different hospitals and universities, surgery has been taught in a theoretical and practical way inside the operating room (OR), with the help of senior surgeons who give the trainees the opportunity of “learning while doing”. This method, though widely used , is now being challenged due to the development of training in new techniques and technologies (laparoscopy and robot-assisted laparoscopy for example) (Cauraugh et al., 1999; Subramonian et al., 2004). There is also enough evidence that undergoing specialised courses for training in laparoscopy helps the trainee to obtain basic knowledge in laparoscopic psychomotor and theoretical skills (Simons et al., 1995; Katz 2006; Ghomi et al., 2007; Ascher-Walsh , et al., 2007; Botchorishvili , et al., 2012).

Many scientific societies today encourage and require surgeons to have adequate training and theoretical knowledge before performing actual OR surgeries. This is also the case in minimally invasive surgery (MIS) in gynaecology. The European Society for Gynaecological Endoscopy (ESGE) has developed a “step by step” Certification and Diploma Programme, the Gynaecological Endoscopic Surgical Education and Assessment (GESEA) with different levels according to each participant's knowledge and laparoscopic skills (Tanos et al., 2016; Campo et al., 2016). This programme certifies gynaecologists according to their theoretical and psychomotor capacities.

In Clermont Ferrand, France, the Centre International de Chirurgie Endoscopique (CICE) is one of the 11 GESEA Certification accredited centres. Every year the CICE offers courses for French obstetrics and gynaecology (OB GYN) residents and organises the Annual Franco-phone GESEA Diploma Course. All participants are given the test for the Level 1 GESEA Certification in MIS - Bachelor in Endoscopy.

Trainees have performed well in the different CICE courses. Botchorishvili et al. (2012) demonstrated a significant improvement in those who took part in the “Residents’ Course”. The same findings can be observed when evaluating the evolution and results of the participants of the Annual Diploma Programmes. But until now, as far as we know, there has been no publication comparing results and evolution of both groups.

The objective of this study was to evaluate the impact of the laparoscopic component of the GESEA Training and Certification courses in two different populations at CICE. The two populations were (a) French Residents in OB GYN who undertook the Residents’ Course and (b) those OB GYN specialists who undertook the Annual GESEA Diploma Course at CICE.

## Materials and methods

### Participants

This study included all the participants attending the CICE Residents’ Course and the Annual Francophone GESEA Diploma Course during 2019. At the beginning of each course, participants were asked to state if they had previous endoscopic surgery experience (defined as at least 30 “level 1” endoscopic surgeries performed as first surgeon) and/or experience with “hands-on” laparoscopic training exercises.

### Description of the Courses

Residents’ Course and the Annual Diploma Course were conducted 2 months apart. The Residents’ Course consisted of two 3-day long modules and the Diploma Course, two 5-day long modules. As described in Botchorishvili et al. (2012) they are both based on a structured curriculum, with theoretical lessons (basic knowledge of anatomy, energies, ergonomics, placement of trocars, operative techniques) and practical sessions of laparoscopic suturing on pelvic-trainers and live animal surgery (performing laparoscopic pig nephrectomy). The course also gave the opportunity to train in some of the Certification Level 1 Exercises comprising Laparoscopic Skills Training and Testing model (LASTT), and the Suturing Training and Testing model (SUTT- detailed below). The final component of the course was to take the Certification theoretical and practical test for Level 1. The Residents’ Course Programme consists of 18 hours of theory and 36 of practical training (12 hours in suture exercises with a pelvic-trainer simulator and 14 hours in live animal surgery).

The Diploma Course Programme consists of 45 hours of theory (including 8 hours of transmitted live surgery from the OR) and 24 hours of practical training consisting of 20 hours in suture exercises with a pelvic-trainer simulator and 4 hours in live animal surgery. In both programmes, practical activities are supervised by certified tutors. For the suture training, each participant works with one partner and for every 4 to 6 participants there is one tutor. For the animal surgery, trainees also work in pairs, and for every 2 pairs there is one certified tutor to help and guide.

The Level 1 Certification Exercises (LASTT and SUTT) consists of standardised training and testing models with different goals and objectives (European Board and College of Obstetrics & Gynaecology (EBCOG), 2014).

### Laparoscopic Exercises

As explained in Campo et al. (2016) LASTT has 3 different exercises: 1) LCN (Laparoscopic Camera Navigation), where the objective is to recognise different predefined characters, in less than 120 seconds using a 30° optic. 2) HEC (hand-eye coordination); the objective is to use the camera and laparoscopic forceps correctly to place 6 different colour rings in a predefined location in less than 180 sec. 3) BMC (bimanual coordination): here the objective is to place 6 different coloured pins in a predefined location in less than 180 seconds, using both hands and passing the pin from one forceps to the other one before placing it in its defined location. The SUTT test challenges participants to achieve, in 15 minutes, 5 correct stitches, performing an intra-corporeal surgeon's square knot with a locking sequence and avoiding (if possible) any trauma.

Pelvic-trainer sessions are performed using a previously validated video-trainer suturing model (Botchorishvili et al., 2012) similar to that used in the MISTELS (McGill Inanimate System for Training and Evaluation of Laparoscopic skills) skill set (Karl Storz, GmbH & Co, Tuttlingen, Germany;Korndorffer et al., 2005; Derossis et al., 1998). The task involves the placement of a 12 cm suture through premarked points in a longitudinally incised chicken leg. The suture is then tied using an intra-corporeal surgeon’s square knot (Derossis et al., 1998).

The animal surgeries (porcine laparoscopic nephrectomies) are performed using general anaesthesia, and in accordance with French law on care and use of laboratory animals and the European Community Guidelines for the use of experimental animals. The goal is to perform a complete nephrectomy, using standard instruments, in a realistic tissue model (Botchorishvili et al., 2012).

### Timing of Evaluations

During both courses, participants had 3 evaluations for the LCN (initial day, the day before ending the course and the certification day) and 2 evaluations for HEC, BMC and SUTT (initial day and the certification day).

### Statistics

Statistical analyses were performed using Stata software, version 13 (StataCorp, College Station, TX, US). The tests were two-sided, with a type I error set at 5%. Continuous data were collected and summarised in the form of either arithmetic mean ± standard-deviation (SD), or median and interquartile range, according statistical distribution. The assumption of normality was assessed by using the Shapiro-Wilk test. Random-effects models (linear regression) for correlated data were performed to analyse repeated data more precisely to evaluate, when appropriate, time-point evaluation and group (course: Residents, Diploma, and Diploma MOD) effects and their time x group interaction, taking into account between and within participant variability (subject as random-effect). The Gaussian normality of residuals from these models were studied using the Shapiro-Wilk test. When appropriate, a logarithmic transformation has been used to approximate a normal distribution. Concerning non-repeated measures, categorical parameters were compared between groups using chi-squared or Fisher’s exact tests, whereas continuous variables were compared by analysis of variance (ANOVA) or Kruskal-Wallis test when assumptions of ANOVA were not met. The homoscedasticity - variance along the regression line -was studied using the Bartlett’s test.

## Results

During 2019, one Annual Diploma Course and four French Residents’ Courses were held in CICE. The Annual Diploma had 69 participants; 29(42%) from different French regions, 32 (46%) from other Francophone countries and the remaining 8 (12%) from Germany, Albania and Switzerland. The Residents’ Courses had a total of 57 French participants.

In the Annual Diploma group, 34 participants (49.3%) were OB GYN specialists and the other 35 (50.7%) were residents in OB GYN. The residents of this group were mostly non-French (only 12/35 (34%) were French). With the intention of observing if there were differences between the residents and the OB GYN specialists within the group, we made a subgroup (‘Diploma MOD’), where we analysed only the 34 OB GYN specialists taking part in the Annual Diploma (excluding the residents).

The main characteristics of the groups are described in Table I. The mean age was lower in the Residents course, 28.4±1.6 versus 35.2±8.0 years (p<0.001), and they had a statistically significant higher “previous experience” in both laparoscopy (LPC) and hysteroscopy (HCP) (p<0.001). In Table II the previous experience of all participants in “hands-on” activities relevant to endocopic surgery including animal model surgery, training box, experience with virtual reality and use of video games is shown. French Residents consistently had more experience with animal model surgeries and training box exercises (p<0.001) than all those in the Annual Diploma. Furthermore, residents of the Annual Diploma Course have greater familiarity with video games compared with the French Residents and the OB GYN specialists of the Annual Diploma Course (p<0.001).

**Table I:** Main characteristics of the participants.

Course	No. of participants	Mean age, years (range)	Male/Female	Previous experience LCP	Previous experience HCP	Dominant Hand (Right/Left)
Residents	57	28 (25-34)	10 / 47	42.0%	59.6%	49 / 8
Diploma	69	35 (26-59)	25 / 44	36.2%*	40.6%*	65 / 4
Diploma MOD	34	40 (30-59)	8 / 26	32.4%*	41.2%*	34 / 0

*All results statistically significant compared to Residents Group (p<0.001), LCP: Laparoscopy, HCP: Hysteroscopy

**Table II:** Exposure to hands-on activities relevant to endoscopic training.

Course	Animal Model	Training Box	Virtual Reality	Other Psychomotor skills
Residents	67%	93%	32%	18%
Diploma	36%*	67%*	26%*	26%*
Residents of Diploma Course	40%*	80%*	29%	37%**

*All results statistically significant compared to Residents Group (p<0.001)

**Statistically significant compared to Residents Group and OB GYN specialists in Diploma Course (p<0.001))

**Figure 1:**
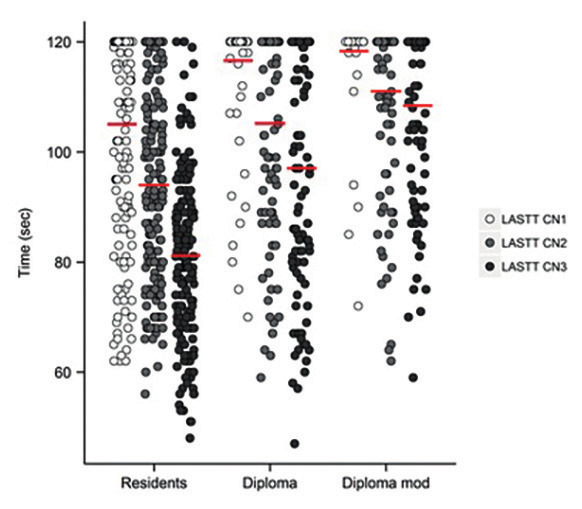
— Box plot of Mean Results and Progression of LASTT camera navigation (CN) exercise in all groups (IC 95%).

The results of three different LASTT LCN exercises (first a basic evaluation, a second one before the end of the course, and the final evaluation – certification test) are given in Figure 1. The difference between those on the Residents’ Course and those undertaking the Diploma Course was statistically significant in all evaluations (105±19 vs 117±9 seconds, 94±18 vs 108±17 seconds and 81±15 and 103±20 seconds Respectively, p<0.001). Comparing how many participants successfully completed the exercise, we found that for the first attempt 53.7% of the Resident Group and 13.2% of the Diploma Group (p<0.001) were successful. This increased in the final attempt to 96% and 59.7%, respectively (p<0.001).

**Figure 2:**
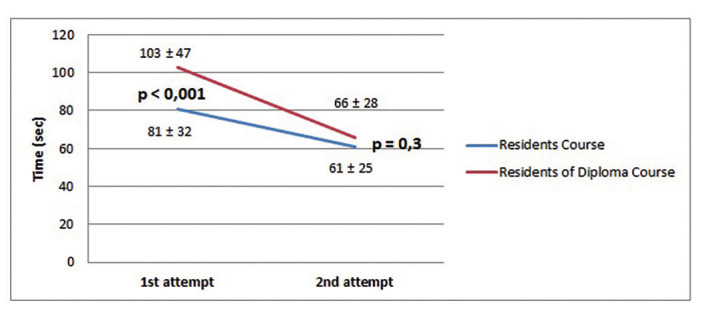
— Results and progression of hand-eye coordination (HEC) exercise.

**Figure 3:**
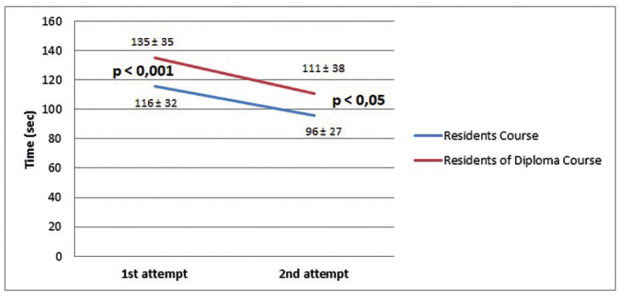
— Results and progression of bimanual coordination (BMC) exercise.

The participants performed the HEC and the BMC exercises only twice, once at the beginning of their assignment and again at the end for their certification test. The results were as follows; for Residents versus Diploma for HEC: 81±32 vs 108±46 seconds for the first exercise and 61±25 vs 76±33 seconds for the certification test (p<0.001); and for the BMC exercise: 116±32 vs 141±35 seconds and 96±27 vs 122±41 seconds (p<0.001), respectively. In both groups we found a significant improvement when comparing the first and second attempts (p<0.001). In The results of the Residents ´ group versus the participants of the Diploma Course Group are presented in Figures 2 and 3.

For the SUTT exercise (also performed twice), the results are given in Table III. For the ‘Analysing Time’ segment, in the first evaluation we observed a significant difference between the results for Residents’ and Diploma Course p<0.001 (this was even more pronounced compared with the Diploma MOD). In the second evaluation, Residents had an increase in time (from 647±133 to 756±126 sec, p<0.001) and Diploma participants had a significant improvement (from 846±122 to 819±116 sec, p<0.001). When observing the amount of correct stitches, the first evaluation showed a significant difference between Residents and Diploma (p<0.001). But in the second evaluation this difference disappeared and both groups had in average of 4 stitches correctly completed; p=0.48). However, a significant difference remained between Residents and Diploma compared to participants of Diploma MOD (p<0.001 in each case). Regarding the ability to produce a correct knot, the Residents had a better performance (maximum score) in the first evaluation, compared with the Diploma group (p<0.001) but in the second evaluation, both groups obtained the maximum score with no statistical differences (p=0.07). Finally, we found no differences when we evaluated the trauma in the stitching model at the end of each exercise (p=0.19 and p=0.63 respectively).

**Table III:** – Suture exercises results.

COURSE	SUTT1 Time	SUTT1 Stitch	SUTT1 Knot	SUTT1 Trauma	SUTT2 Time	UTT2 Stitch	SUTT2 Knot	SUTT2 Trauma
Residents	647± 133 sec	5	2	1	755± 126 sec	4	2	1
Diploma	846± 122 sec*	3*	1*	1	791± 116 sec*	4	2	1
Diploma MOD	868± 85 sec*	3*	1*	1	852± 92 sec*	3**	2	1

* All results statistically significant compared to Residents Group (p<0.001)

**Statistically significant compared to Residents Group and Diploma (p<0.001)

Finally, of all the participants, only 2 failed to pass the Certification Exam, and both of them were in the Diploma group (specifically in the Diploma MOD group) (p<0.001).

## Discussion

There is much published evidence on training courses in surgery, and many of them involve Residents’ Courses (Cundiff et al., 1997; Essani et al., 2009). However, there is less reported evidence comparing residents with more experienced surgeons. Boza et al. (2017) conducted a study where they compared the performance of younger residents, who had previously undertaken a complete simulation programme, and experienced surgeons (without any simulation exercises) in practical OR surgery. They demonstrated that after systematic training, the younger residents obtained comparable, and even better, results than senior surgeons. Furthermore, they found that an appropriate training programme enables the trainee to transfer the skills to the OR. In CICE there is a deep commitment to performing and enhancing the courses that are offered to the medical community because we are convinced that the best way to be prepared for actual OR surgery is appropriate and focussed previous training.

Our study results revealed that the Annual Diploma Course participants (Residents and specialists in OB GYN) had less previous experience than the French Residents in (a) the practice of endoscopic surgery, (b) in previous animal model surgery and (c) in the use of laparoscopic training boxes. Residents normally have more experience in laparoscopic and hysteroscopic procedures during their training period so we expected them to have better results than older OB GYN specialists. However, it is also notable that French Residents have more “hands-on” lab training than non-French Residents in the Annual Diploma Group. These differences between Residents’ Formation Programmes in different countries are consistent with existing evidence (Van Kerrebroeck H et al., 2015).

Previous experience in MIS and laparoscopic training is reflected in the results of the different tests this article analyses. The French residents had consistently better results in all tests, except in the second evaluation of SUTT, where we observed an increase in the time they took to complete the exercise. However, we consider this not overly significant because they all finished the exercise correctly in the time given (less than 15 minutes) together with good scores on the other features we evaluated. In addition, all results were more significant when comparing the residents with the Diploma MOD participants (this means excluding the residents that attended the Annual Diploma Course). In fact, we found that residents taking part in the Diploma Course had consistently better results than the specialists in OB GYN participants of the same group.

A further significant result is that at the end of the course, participants of the Annual Diploma Course obtained similar results to those obtained by the French Residents (Residents' Group) in their first attempt. This may be encouraging for senior and/ or older gynaecologists because it may mean that training improves their surgical techniques and updates some psychomotor skills.

We also noticed that in both groups that we studied the improvement shown in all exercises was significant. This probably is due to several factors, but we think that the sum of theoretical knowledge, training with simulators and live animal surgery, combined with a repetition of some standardised exercises, helps the trainee obtain skills necessary to be confident and work safely during simulated laparoscopic procedures. In addition this shows that, for all surgeons, the undertaking of regular training courses helps to develop and enhance psychomotor skills and abilities, which could then be transferred to the OR (Campo et al., 2012).

Finally we note that although two participants of the Diploma course failed the Certification Test because of low results in the practical exercises: this is not a high failure rate compared with the number who passed the test. This may be considered confirmation that the certification proposed by GESEA offers a filter for those who do not have the adequate skills to perform MIS.

This study has some limitations. One of these is we were unable to compare two radically different groups. This was because almost half of the Diploma participants were residents. However, this gave us the opportunity to compare French Residents with Non-French residents and residents with specialists in OB GYN. The sub-analysis of only the OB GYN specialists in the Annual Diploma Course group helped demonstrate these differences.

Another limitation is that not all exercises were performed the same amount of time although the camera navigation (LCN) exercise was repeated 3 times. The evaluation of only 2 repetitions may place a notable constraint on our results.

Finally, even though both courses are very similar in their structure, the Residents’ Course has more training hours (35 vs 25 hours) and less theoretical exposition (18 vs 45 hours), so this can further constrain the results. Conversely the first evaluation of every exercise was at the beginning of each course, and even at that time we could already see an important difference in the performance of two groups.

## Conclusions

Despite the limitations noted above, we consider our study useful because it confirms that residents with previous training in MIS and laparoscopy compared to older OB GYN specialists achieve better results in all exercises we evaluated. Also, significant differences were observed when comparing French residents with non-French residents who took part in the Annual Diploma Course, the former having greater experience in laparoscopy and obtaining better results in the tests we evaluated.

We found that both groups showed significant improvement in their test results after attending each course. This encourages us to continue preparing and offering high level training courses for residents and specialists.

Whilst we cannot confirm that all skills learned in these training courses can be fully transferred to the OR, we strongly believe that learning basic theoretical concepts together with practical training will help to enhance the surgeon’s confidence in performing endoscopic procedures in a progressive and safer way.
